# Cyclopedia of the Diseases of Children

**Published:** 1889-11

**Authors:** 


### Book Reviews.

**Cyclopedia of the Diseases of Children—Medical and Surgical.** The articles written especially for the work by American, British, and Canadian authors. Edited by JOHN M. KEATING. Vol. II. Illustrated; 8vo, pp. xii., 1066. Philadelphia: J. B. Lippincott Co. 1889.

The favorable opinion expressed in the August number of the JOURNAL, on the appearance of the first volume of this work, is fully maintained by the study of this second volume, and we can but reaffirm that opinion here. In calling attention to the merits of the work, a critical and complete review of each contribution seems to be out of place. There are no less than sixty-seven separate articles in the present volume, and it will be impossible, in the space at command, to notice them all. By calling attention to a few of them, the general high character of the work may be indicated, and that will accomplish our purpose. The present volume is divided into five parts. Part I. considers the Diseases of the Skin, and is very full and complete. As the care of the skin in infancy is of great importance, so also is a knowledge of its maladies. As far as this can be taught from books, we can find that knowledge here. Of special interest are the articles on Nevus, or Birth-Mark, by Lewis S. Pilcher, of Brooklyn, and on Syphilitic Skin Affections, by J. E. Atkinson, of Baltimore.

Part II. deals with Constitutional Diseases, and Diseases of Nutrition. We find here articles on Scrofulosis, Tuberculosis, Syphilis, Rachitis, Scurvy, Cretinism, The Urinary Diatheses, and Diabetes Mellitus.

The importance of a clear understanding of these subjects cannot be over-estimated, and this entire part will well repay careful study. Particular attention is directed to the articles on Scrofulosis, by Henry Ashby, of Manchester; on Tuberculosis, by A. Jacobi, of New York; on Rachitis, by Thomas Barlow, of London, and Judson S. Bury, of Manchester; and on the Urinary Diatheses, by the late J. Milner Fothergill. We understand from the editor that this was Dr. Fothergill's last contribution to medical literature, and that his death was announced a few days after the manuscript was received. It considers the subject under three heads, viz.: Oxaluria, Phosphaturia, and Lithuria. Its introduction is as follows: "This article is an attempt to gather together what is known of an interesting subject not nearly so carefully studied as it was half a century ago, and as it probably will be less than half a century hence. On the first two matters, our knowledge is in a fragmentary condition, especially as to oxaluria. On the last subject, we are in possession of considerable knowledge." His conclusions in regard to oxaluria are thus presented: "It is rather a matter of scientific curiosity, with its octahedral and dumb-bell crystals, than of clinical value, and some excellent works on diseases of children say nothing about oxaluria. The sort of child most likely to present it is that to be described at some length in the section on Lithuria, and the reader will find that its associations are those of systemic debility." Further on, in the course of his remarks on Phosphaturia, it is stated that "A great deal has been written and said about phosphatic deposits, but we seem to know very little more than what we find in the urine, and its behavior; and this has more interest for the curious inquirer than practical value for the physician. The views promulgated by Prout have not stood the test of time." These quotations will serve to express the present state of our knowledge on these two subjects. With lithuria, however, it is different. Although Dr. Fothergill's views on lithuria are well known, it will repay any one to study his latest words upon it. Starting from the point that "Lithogenesis is Reversion," he considers it as to its etiology, its diagnosis, its pathology, and its treatment. We cannot follow him, at this time, through these interesting pages, and have space but for his general conclusion, viz.: "The subject can easily be summed up. The newly-born child possesses the uric-acid formation as a normal matter. But it gradually outgrows or rises above this lowly formation, and leaves it behind at puberty, *i. e.*, if it is equal to the urea-formation. A delicate child fails to achieve this, and it then becomes our duty to give it the requisite help, if we possibly can, by the application of the principles just laid down."

Part III. treats of the Diseases of the Respiratory Tract, and opens with a classic paper on Nasal Obstruction, by John N. Mackenzie of Baltimore. If the general physician would study the principles here inculcated, he would appreciate, more than is apparent at present, the disastrous consequences to the organs of respiration, audition, and voice-production that may arise from the obstruction of the nasal passages. He says, truly, that but few of the older writers recognized the complications occurring from obstruction of the nostrils, and that even now the full recognition of these evils is limited principally to those whose special studies have led them in this direction. And again, "Many an aural catarrh has been allowed to end in hopeless deafness, many a naso-laryngeal inflammation has become inveterate and incurable, from failure to recognize the evils which result therefrom; and were the statistics of such cases carefully compiled, they would appear to many in the form of a revelation."

Following this article, we find considered in their order the various diseased conditions as seen in the nose, in the pharynx, in the larynx, and in the lungs. These are studied with special reference to their occurrence in children. So far as our knowledge extends, this is the only place where such a collection of essays can be found. Thirty-two out of the sixty-seven articles in the volume are found in this part, which will indicate, to some degree, the thoroughness of the work. To appreciate it fully, each article must be investigated by the reader himself.

Part IV. presents the Diseases of the Circulatory, Hematopoietic, and Glandular Systems. The opening article is on Functional Disorders of the Heart, by J. M. Da Costa, of Philadelphia. Following this, the Congenital Affections of the Heart are considered by William Osler, of Baltimore. This article is finely illustrated, and is of great practical importance. Special mention, also, should be made of the essays on Endocarditis, Acute and Subacute, by W. B. Cheadle, of London, and Myocarditis and Cardiac Aneurism, by J. Mitchell Bruce, also of London. The last one contains some superb illustrations, and cannot fail to be appreciated.

The title of Part V. is Diseases of the Mouth, Tongue, and Jaws. The first article, upon the Diseases and Care of the Teeth, is from the pen of Edwin T. Darby, of Philadelphia, and contains many suggestions which will be of assistance in the treatment of these organs. Dr. Roswell Park, of this city, contributes the next article, upon the Congenital Defects and Deformities of the Face, Lips, Mouth, Tongue, and Jaws. Like all Dr. Park's work, it is concise, and, at the same time, thoroughly well done. He begins with some brief considerations of the embryology of the parts, and then presents in detail the deformities seen in each of the organs mentioned. Many illustrations add to the value of the article. The other articles in this part are: Diseases of the Mouth, by W. W. Allchin, of London; Hare-lip and Cleft-palate, by J. Ford Thompson, of Washington; and Injuries and Diseases of the Jaws, by J. Ewing Mears, of Philadelphia.

We trust enough has been said to indicate the high character of the volume, and to stimulate all those who desire the latest thought of the profession on the subjects upon which it treats to become students of its pages.

It is a credit to the publishers, to the editor, and to the various contributors. F. H. P.

